# Novel synthetic co‐culture of *Acetobacterium woodii* and *Clostridium drakei* using CO_2_ and in situ generated H_2_ for the production of caproic acid via lactic acid

**DOI:** 10.1002/elsc.202100169

**Published:** 2022-05-22

**Authors:** Jan Herzog, Alexander Mook, Lotta Guhl, Miriam Bäumler, Matthias H. Beck, Dirk Weuster‐Botz, Frank R. Bengelsdorf, An‐Ping Zeng

**Affiliations:** ^1^ Institute of Bioprocess and Biosystems Engineering Hamburg University of Technology Hamburg Germany; ^2^ Institute of Microbiology and Biotechnology Ulm University Ulm Germany; ^3^ Department of Energy and Process Engineering Chair of Biochemical Engineering Technical University of Munich TUM School of Engineering and Design Garching Germany

**Keywords:** bioelectrochemical system, carbon fixation, cell–cell interaction, constraint‐based modeling, in situ electrolysis

## Abstract

*Acetobacterium woodii* is known to produce mainly acetate from CO_2_ and H_2_, but the production of higher value chemicals is desired for the bioeconomy. Using chain‐elongating bacteria, synthetic co‐cultures have the potential to produce longer‐chained products such as caproic acid. In this study, we present first results for a successful autotrophic co‐cultivation of *A. woodii* mutants and a *Clostridium drakei* wild‐type strain in a stirred‐tank bioreactor for the production of caproic acid from CO_2_ and H_2_ via the intermediate lactic acid. For autotrophic lactate production, a recombinant *A. woodii* strain with a deleted Lct‐dehydrogenase complex, which is encoded by the *lctBCD* genes, and an inserted D‐lactate dehydrogenase (LdhD) originating from *Leuconostoc mesenteroides*, was used. Hydrogen for the process was supplied using an All‐in‐One electrode for in situ water electrolysis. Lactate concentrations as high as 0.5 g L^–1^ were achieved with the AiO‐electrode, whereas 8.1 g L^–1^ lactate were produced with direct H_2_ sparging in a stirred‐tank bioreactor. Hydrogen limitation was identified in the AiO process. However, with cathode surface area enlargement or numbering‐up of the electrode and on‐demand hydrogen generation, this process has great potential for a true carbon‐negative production of value chemicals from CO_2_.

**Abbreviations**
AiO: all‐in‐one electrode
BES: bioelectrochemical system

## 1 INTRODUCTION

With the climate crisis progressing due to massive carbon dioxide emissions of almost all major industries [1], a pressing issue is to avoid further CO_2_ emissions while at the same time reducing the CO_2_ concentration in the atmosphere. Up to this day, the chemical industry is the third‐largest industrial sector concerning direct CO_2_ emissions [2]. The integration of CO_2_ in the production processes for carbon‐based chemicals would be a substantial step toward a non‐fossil‐based economy while reducing CO_2_ concentrations in the atmosphere [3]. One high‐value chemical which is still produced mainly from petrochemicals is caproic acid. It has a variety of applications such as flavor additive in the food industry, antimicrobial agent in the pharmaceutical industry, or possibly as precursor in the production of biofuels, among others [4]. Replacing the conventional production of chemicals such as caproate with carbon‐neutral or even carbon‐negative processes will be a crucial step toward a climate positive future.

*Acetobacterium woodii* is one of the most studied acetogens and its central carbon fixation pathway, the Wood–Ljungdahl pathway (WLP), allows *A. woodii* to subsist on a variety of C1 carbon substrates such as CO_2_/H_2_, syngas [5], methanol [6], and formate [7]. The availability of genetic tools for this model organism [8, 9] lends itself to strain engineering for novel products. Recently, *A. woodii* was engineered to produce lactate from CO_2_ and H_2_ [10] by exchange of the native lactate consuming lactate dehydrogenase with a D‐lactate dehydrogenase from *Leuconostoc mesenteroides* subsp*. mesenteroides* encoded by the *ldhD* gene [11]. Lactate produced by the mutant *A. woodii* [P*_bgaL_* _*ldhD*_NFP] can be used as feedstock for a variety of strains in co‐cultivation to generate value‐added products [12, 13]. One possible partner for co‐cultivation is the obligate anaerobe *Clostridium drakei*, first described as *Clostridium scatologenes* SL1 [14, 15], which is able to grow with diverse substrates including lactate. *C. drakei* possesses the *bcd*/*hcs* gene cluster and the respective enzymes are responsible for the elongation of acetyl‐CoA to butyryl‐CoA and subsequently hexanoyl‐CoA, enabling the production of short and medium‐chain organic acids such as butyrate and caproate [16].

Co‐cultivation faces its own set of challenges, for example, cultivation conditions and supply of substrate(s) for optimal growth and productivity of both species. While CO_2_ as the carbon source for the sustainable fermentation with acetogens is abundant, hydrogen needed as electron donor presents some issues concerning safety and climate‐friendly production. In 2020, about 99% of the worldwide produced H_2_ came from fossil‐based sources such as coal and natural gas, while carbon‐low production technologies such as water electrolysis or conventional production coupled with carbon capture, utilization, and storage (CCUS) techniques made up only 0.73%. The production of H_2_ accounts for 2.5% of global CO_2_ emissions in energy and industry. [17] While electrolytic H_2_ production is estimated to grow up to 5 Mt per year in 2030 [17], this technology will still need an electric supply from renewable sources to be carbon neutral. Although the amount of energy from renewable sources grows every year [18], their output is not constant and varies with location and conditions such as weather. Therefore, storage of excess energy during peak times is key for a successful energy transformation [19]. Next to accumulators and large pump‐storage plants, electrochemical bioprocesses offer an alternative method of energy conservation in supporting microbial bioconversion [20, 21, 22].

**PRACTICAL APPLICATION**

Global carbon emissions need massive reductions to reach climate goals and limit global warming. One substantial step to achieve this is the utilization of CO_2_ as feedstock to produce value chemicals. This study presents the proof‐of‐concept and preliminary results for a novel and sustainable autotrophic coculture bioprocess to produce the medium‐chain carboxylic acid caproate from CO_2_ and in situ generated H_2_. In particular, we show the successful cultivation of an engineered *Acetobacterium woodii* mutant and a *Clostridium drakei* strain in a synthetic coculture at laboratory scale. Contrary to most bioelectrochemical systems, the electrode used in this study to generate H_2_ in situ has the potential for industrial application given that it is easily scalable. Furthermore, the process allows the regulation of the autotrophic growth via on‐demand H_2_ generation, based on online data such as lactate concentration, which would allow an efficient production of caproic acid from CO_2_.

Given that standard fermentation media are water‐based, a process that would be truly sustainable and carbon‐neutral is the in situ production of H_2_ during the fermentation via electrolysis using electricity from renewable sources. While there are many different options for bioelectrochemical systems (BES) [23], many are hardly scalable and, therefore, unsuited for large scale industrial applications. The process presented here uses the All‐in‐One electrode (AiO) to generate H_2_ in situ and on‐demand. The AiO‐electrode was designed to be easily integrated into any standard bioreactor, thus turning them into BES. The fermentation broth then serves as the working electrode chamber while the counter electrode chamber is found on the inside of the rod‐shaped electrode. This electrode is not only flexible and shows high Faraday efficiencies of up to 80%, but it also has the potential to be scalable for industrial purposes [24, 25, 26].

In this study, we demonstrated that cell growth and lactate production with the *A. woodii* [P*_bgaL_* _*ldhD*_NFP] mutant strain is possible using the AiO‐electrode for in situ H_2_ production. We compared the AiO BES process with conventional gas fermentations to identify the limitation of the present BES process. Furthermore, we present a novel synthetic co‐culture of *A. woodii* [P*_bgaL_* _*ldhD*_NFP] and *C. drakei* to produce caproate employing CO_2_ and in situ generated H_2_ in a batch process. This process shows the potential of a truly sustainable and carbon‐negative process for value chemical production.

## 2 MATERIALS AND METHODS

### 2.1 Microorganism and medium

The strains used in this study are listed with their relevant features in Table 1. Construction of *A. woodii* [P*_bgaL_* _*ldhD*_NFP] and *A. woodii* P_*tet*__*ldhD*_CI_ are described in Refs. [10] and [8], respectively. The *C. drakei* wild‐type strain was obtained from the German Collection of Microorganisms (DSMZ 12750). For the cultivation of all *A. woodii* strains in serum bottles, the modified DSMZ medium 135 as described by Hoffmeister et al. [9], was used (see Tables S1–S4). For bioreactor fermentations, this medium composition was adapted by replacing 0.2 g L^–1^ NH_4_Cl with 0.7 g L^–1^ (NH_4_)_2_SO_4_ and reducing the NaHCO_3_ concentration to 5.0 g L^–1^ as well as the Na_2_S concentration to 0.1 g L^–1^. The medium was always supplemented with 20 μg mL^–1^ uracil.

**TABLE 1:** Bacterial strains used in this study with their relevant features

**Strain**	**Genotype**	**Features**	**Source**
*A. woodii* WT	*Acetobacterium woodii WB1*	Wild‐type *A. woodii* (DMSZ 1030)	DSMZ
*A. woodii* P*_tet__ldhD*_CI_	*Acetobacterium woodii ΔlctBCD ΔpyrE*::*pyrE‐*P*_tet_‐ldhD*	Recombinant *ldhD* from *Leuconostoc mesenteroides* subsp. M*esenteroides* ATCC 8293, anhydrotetracycline (atc) inducible P*_tet_* promoter, chromosomal integration mutant with reconstituted *pyrE* (Awo_c16210), deletion of the native lct‐dehydrogenase complex encoded by *lctBCD* (Awo_c08710, c08720, c08730)	[8]
*A. woodii* [P*_bgaL__ldhD_*NFP]	*A. woodii ΔlctBCD ΔpyrE* [p83_P*_bgaL_* _NFP]	Recombinant *ldhD* from *Leuconostoc mesenteroides* subsp. *Mesenteroides* ATCC 8293 codon optimized for *A. woodii* and N‐terminally fused to FAST, lactose inducible P*_bgaL_* promoter, plasmid‐based mutant with pMTL83251 as backbone plasmid, deletion of *pyrE* (Awo_c16210), deletion of the native lct‐dehydrogenase complex encoded by *lctBCD* (Awo_c08710, c08720, c08730)	[10]
*C. drakei*	*Clostridium drakei SL1*	Wild‐type *C. drakei* (DSMZ 12750)	DSMZ

### 2.2 Serum bottle pre‐culture fermentation

The precultures for all *A. woodii* strains as well as *C. drakei* were cultivated heterotrophically at 30°C using fructose (55.5 mM) and the previously described medium in non‐agitated anaerobic serum bottles. Lactate was added to the *C. drakei* precultures in a 75:25 ratio (g L^–1^) with fructose. The precultures for both strains were incubated for 33–36 h.

### 2.3 Stirred‐tank reactor fermentations with AiO‐electrode

All batch fermentations with the AiO‐electrode were carried out in a 2.0 L stirred‐tank bioreactor (KSF2000, Bioengineering AG, Wald, Switzerland) with a working volume of 1.4 L. Before each fermentation, the medium was sterilized in situ at 121°C for 20 min. Afterwards, the cultivation medium was degassed with N_2_ to ensure anaerobic conditions. An oxygen reduction potential (ORP) sensor was installed to ensure an ORP value lower than –280 mV before inoculation. The pH of the culture was measured via a pH sensor and controlled at pH 7.0 ± 0.2 by the addition of a 5 M KOH solution. The necessary H_2_ for the fermentation was supplied by the AiO‐electrode [26] via in situ electrolysis. The electrode material was platinized titan (platin coating thickness d = 2.5 μm, coating density ρ = 50 g m^–2^); working and counter electrode were separated by a ceramic separator and the O_2_, which was generated during electrolysis in the counter electrode chamber, left the reactor through an exhaust duct. The working electrode surface of the AiO‐electrode was 74.8 cm^2^ while the counter electrode surface was 13.8 cm^2^ (a scheme is shown in Figure S1). The AiO‐electrode was controlled chronopotentiometrically by a potentiostat (Interface 1000, Gamry, Philadelphia, USA) and was operated without reference electrode to maintain a constant electrical current of 600 mA (*j* = 8 mA cm^–2^, *E*_cell_ = 4.2 ± 0.3 V, P = 2.5 W). The medium was saturated with CO_2_ before inoculation and then CO_2_ sparging was turned off for 10 h to ensure a H_2_/CO_2_ ratio of about 70/30%. Subsequently, the CO_2_ gas flow was set to 0.9 L h^‐1^. The culture was agitated with three Rushton disk turbines at an initial stirrer speed of 800 rpm which was increased to 1,000 rpm after 10 h (P V^–1^ = 2.4 – 4.6 W L^–1^). The temperature was controlled at 30°C. For *A. woodii* [P*_bgaL_* _*ldhD*_NFP] cultivations, the production of lactate was induced with the addition of 1 mM lactose. *A. woodii* P_*tet*__*ldhD*_CI_ cultivations were induced with the addition of 0.1 g L^–1^ anhydrotetracycline. Induction was conducted when the batch culture had reached an optical density measured at 600 nm (OD_600_) of 0.79 ± 0.09. For co‐culture fermentations, *A. woodii* [P*_bgaL_* _*ldhD*_NFP] was inoculated first and grown until stationary phase to ensure sufficient production of lactate. Then, 83 mL of a *C. drakei* culture (OD_600_ = 1.75 ± 0.68) from a preculture bottle were added to the bioreactor.

The off‐gas was monitored constantly with a mass flow meter (EL‐FLOW prestige, Bronkhorst High‐Tech B.V., Ruurlo, Netherlands) and a mass spectrometer (Omnistar GDS 300, Pfeiffer Vacuum GmbH, Asslar, Germany). The OD of the culture was measured offline with a spectrophotometer (V3000PC, VWR International GmbH, Darmstadt, Germany) at 600 nm. OD measurements were performed in technical triplicates. Organic acid concentrations were determined via high‐performance liquid chromatography (HPLC) with an UV‐ and RI‐detector as well as an Aminex HPX‐87H column (Biorad Laboratories Inc., Berkley, USA). The measurements were run at 30°C with 0.1% trifluoroacetic acid as eluent and a constant flow rate of 0.6 mL min^–1^. The cell dry weight was calculated with a previously determined linear correlation factor of OD_600_ × 0.51 g L^–1^. For the calculation of carbon recovery (C_recovery_), Equation (1) was applied.

(1) $$C_{recovery}=\frac{\sum_{i}^{n}n_{i}\cdot C_{i}^{*}}{\sum_{j}^{n}n_{j}\cdot C_{j}^{*}}$$

where *n_i_* [mol] is the total generated amount of a product compound, and *n_j_* [mol] is the amount of a substrate concentration. C_i_* and C_j_* are the number of carbon atoms of the respective compounds. Lactate, acetate, formate, caproate, butyrate as well as biomass were considered as products while CO_2_, NaHCO_3,_ and in the case of the co‐cultivation, lactate were considered as substrates (see also Equation S1).

### 2.4 Stirred‐tank reactor fermentations with direct H_2_ sparging

Conventional gas fermentations were carried out in a 2.0 L stirred‐tank bioreactor (Labfors, Infors AG, Bottmingen, Switzerland) with a working volume of 970 mL. Before each batch fermentation, the medium and the bioreactor were sterilized at 121°C for 20 min. Afterwards, the cultivation medium was degassed with a gas mixture of H_2_/CO_2_ (70/30%) for 4 h before inoculation to ensure anaerobic conditions and to saturate the medium with the respective gasses. The flow rates of the gas mixture were adjusted to 0.5 and 0.025 vvm, respectively. The pH of the culture was measured via a pH sensor and controlled at pH 7.0 ± 0.1 by the addition of a 3 M NaOH solution. The culture was agitated with two Rushton turbines at 1,000 rpm (P V^–1^ = 6.8 W L^–1^) and the temperature was controlled at 30°C. The batch cultivations with *A. woodii* [P*_bgaL_* _*ldhD*_NFP] were induced with the addition of 1 mM lactose at an OD_600_ of 0.86 ± 0.01. Adjustment of the inlet gas flow, as well as exhaust gas, OD_600_, and product analytics, were carried out as described by Bäumler et al. [27].

### 2.5 Stoichiometric and constraint‐based modeling of metabolic fluxes

CellNetAnalyzer [28, 29] was used in combination with an *A. woodii* core model [30] for constraint‐based modeling of the H_2_/CO_2_ demand for product formation in the AiO‐process and the conventional gas fermentations of *A. woodii* [P*_bgaL_* _*ldhD*_NFP]. The core model, consisting of 117 species and 118 reactions, was modified to accommodate the exchange of the Etf‐coupled lactate dehydrogenase with the recombinant NADH‐dependent LDHD. In addition, the rate constraints for methanol, CO, fructose, and glucose uptake, as well as ethanol export were set to zero to model the intracellular fluxes with H_2_/CO_2_ as sole substrate and energy sources. Formate was modeled as an exported product to accommodate the measured extracellular concentrations. For the calculation of H_2_/CO_2_ demand, the endpoint product concentrations (mol L^–1^) were modeled as secretion rates (mol L^–1^ h^–1^) through two assumptions. Firstly, a metabolic steady state was assumed throughout the fermentation to meet the requirements of constraint‐based modeling [31]. Secondly, the process time was abstracted as 1 h to allow for the conversion of concentrations to rates and vice versa, to implement the respective values in the respective *A. woodii* core model in CNA. The flux scenario was validated with the integrated “Check feasibility of flux scenario”‐function before flux balance analysis was performed. Comparison with measured values was achieved by dividing the measured substrate amount by the respective working volume at the end of the fermentation and forming a ratio with the calculated values for CO_2_ and H_2_.

## 3 RESULTS

### 3.1 *A. woodii* gas fermentations with AiO‐electrode

**FIGURE 1:**
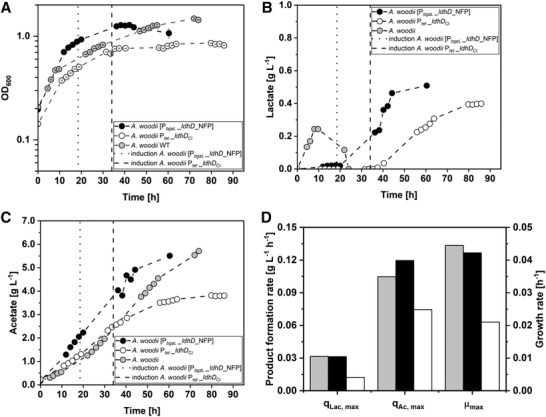
Comparison of autotrophic stirred‐tank batch cultivations of *A. woodii* WT (light gray circles) *A. woodii* [P_*bgaL*__*ldhD*_NFP] (black circles) and *A. woodii* P_*tet*_ *_ldhD*_CI_ (white circles). (A) Optical cell density (OD_600_); (B) concentration of lactate measured in the culture; (C) concentration of acetate measured in the culture; (D) maximum lactate formation rate (q_lac, max_), maximum acetate formation rate (q_ac, max_) and maximum growth rate (μ_max_). The dotted black line indicates the time point of induction of *A. woodii* [P_*bgaL*_ *_ldhD*_NFP] with 1 mM lactose. The dashed black line indicates the time point of induction of A. woodii P_*tet*__ldhD_CI_ with 0.1 g L^–1^ anhydrotetracycline. (T = 30°C; pH = 7.0; P V^–1^ = 2.4–4.6 W L^–1^; F_CO2_ = 0.9 L h^–1^; I_AiO_ = 600 mA; V_0_ = 1.4 L)

First batch experiments were conducted with the use of the AiO‐electrode BES and different *A. woodii* strains. All strains were cultivated separately as described in Section 2.3. The comparison of all autotrophic batch cultivations can be seen in Figure 1. All three strains started growing shortly after inoculation and reached their maximum growth rate during this phase. *A. woodii* [P*_bgaL_* _*ldhD*_NFP] reached a μ_max_ of 0.042 h^–1^ (t = 6.0 h) which was similar compared to the maximum growth rate of *A. woodii* WT (0.045 h^–1^; t = 6.3 h). The *A. woodii* P*_tet__ldhD*_CI_ culture reached a lower μ_max_ of 0.021 h^–1^ (t = 5.5 h). After 18.5 h of cultivation time, the *A. woodii* [P*_bgaL_* _*ldhD*_NFP] strain was induced for lactate production with 1 mM lactose at an OD_600_ of 0.89 ± 0.01. Due to slower growth, the *A. woodii* P*_tet__ldhD*_CI_ strain was induced not until 34.1 h at an OD_600_ of 0.68 ± 0.02. The highest maximum OD_600_ of 1.48 ± 0.03 was reached by the *A. woodii* WT strain after 72.0 h of process time. *A. woodii* [P*_bgaL_* _*ldhD*_NFP] reached a maximum OD_600_ of 1.28 ± 0.05 after 42.2 h. Subsequently, its growth stopped and the OD_600_ started to decline. The *A. woodii* P*_tet__ldhD*_CI_ cultivation reached a maximum OD_600_ of 0.86 ± 0.01 after 79.8 h during the stationary phase. Lactate was produced by all strains during the cultivations. *A. woodii* WT produced a maximum of 0.24 g L^–1^ in the first 8 h of the cultivation with a maximum lactate formation rate of 0.032 g L^–1^ h^–1^ (t = 6.3 h). Subsequently, the lactate was completely consumed by the *A. woodii* WT strain during the autotrophic batch fermentation. *A. woodii* [P*_bgaL_* _*ldhD*_NFP] started producing lactate with a formation rate of 0.011 g L^–1^ h^–1^ after induction. Then, the lactate formation rate increased to its maximum of 0.031 g L^–1^ h^–1^ (t = 40.3 h) and at the end of the cultivation after 60.4 h, a total of 0.51 g L^–1^ of lactate was produced. The *A. woodii* P*_tet__ldhD*_CI_ cultivation reached its maximum lactate formation rate of 0.012 g L^–1^ h^–1^ shortly after induction (t = 49.1 h), producing lactate up until 79.8 h of fermentation time. Afterwards, the lactate concentration stayed constant at a maximum value of 0.40 g L^–1^.

Acetate was produced by all three strains from the beginning of the fermentation. The highest acetate formation rate was reached by *A. woodii* [P*_bgaL_* _*ldhD*_NFP] with 0.12 g L^–1^ h^–1^ (t = 15.0 h), followed by 0.11 g L^–1^ h^–1^ (*t *= 37.5 h) for *A. woodii* WT. During the cultivation of *A. woodii* P*_tet__ldhD*_CI,_ the maximum acetate formation rate added up to 0.08 g L^–1^ h^–1^ (t = 25.3 h). While the acetate production during the *A. woodii* P*_tet__ldhD*_CI_ cultivation started to decline after 60 h, both *A. woodii* [P*_bgaL_* _*ldhD*_NFP] and the *A. woodii* WT strain kept producing acetate at similar rates until the cultivation was terminated. The maximum acetate concentrations added up to 5.5 g L^–1^ with *A. woodii* [P*_bgaL_* _*ldhD*_NFP], 5.7 g L^–1^ with the *A. woodii* WT strain, and 3.8 g L^–1^ with the *A. woodii* P*_tet__ldhD*_CI_ strain.

The overall H_2_ uptake added up to 468.9 mmol with *A. woodii* [P*_bgaL_* _*ldhD*_NFP], followed by 443.3 mmol with *A. woodii* WT and 385.5 mmol with *A. woodii* P*_tet__ldhD*_CI_. The total CO_2_ uptake added up to 229.0 mmol with *A. woodii* [P*_bgaL_* _*ldhD*_NFP], followed by 211.9 mmol with *A. woodii* WT and 97.4 mmol with *A. woodii* P*_tet__ldhD*_CI_. The AiO‐electrode showed a maximum H_2_ production rate of 6.4 ± 1.2 mmol L^–1^ h^–1^ during all three cultivations. Carbon balances were closed within an estimation error of 5%. Details are listed in Table 2. In summary, the *A. woodii* [P*_bgaL_* _*ldhD*_NFP] strain produced 1.3‐fold more lactate and 1.5‐fold more acetate compared to the *A. woodii* P*_tet__ldhD*_CI_ strain.

**TABLE 2:** Maximum product formation and consumption rates, maximum cell density and growth rate, maximum product concentrations, total gas uptake and carbon balances of all autotrophic batch processes with *A. woodii* in stirred‐tank bioreactors applied in this study

Parameter		Unit	WT ‐AiO	P_*tet*_ ‐ AiO	P_*bgaL*_ ‐ AiO	P_*bgaL*_ ‐ high F_Gas_	P_*bgaL*_ ‐ low F_Gas_	Co‐culture
OD_600_	OD_max_	–	1.48	0.86	1.28	2.07	2.26	1.48
	μ_max_	h^–1^	0.04	0.04	0.02	0.07	0.08	0.04
Lactate	c_Lac, max_	g L^–1^	0.24	0.40	0.51	8.07	3.41	0.52
	q_Lac, max_	g L^–1^ h^–1^	0.03	0.01	0.03	0.27	0.19	0.03
	‐q_Lac, max_	g L^–1^ h^–1^	0.01	–	–	–	–	0.01
Acetate	c_Ac, max_	g L^–1^	5.71	3.81	5.34	16.10	17.20	6.28
	q_Ac, max_	g L^–1^ h^–1^	0.10	0.07	0.12	0.46	0.51	0.14
Caproate	c_Cap, max_	g L^–1^	–	–	–	–	–	0.11
	q_Cap, max_	g L^–1^ h^–1^	–	–	–	–	–	0.01
Formate	c_Form, max_	g L^–1^	0.46	0.58	0.87	1.20	1.02	0.67
Butyrate	c_But, max_	g L^–1^	–	–	–	–	–	0.55
CO_2_	$n_{{\mathrm{CO}}_{2},\;\mathrm{total}}$	mol	0.21	0.10	0.23	0.80	0.67	0.23
H_2_	$n_{H_{2},\;\mathrm{total}}$	mol	0.44	0.39	0.47	1.35	1.19	0.47
C‐balance		–	0.97	1.03	1.00	0.95	0.95	0.97

### 3.2 *A*. *woodii* gas fermentations with H_2_ gassing

**FIGURE 2:**
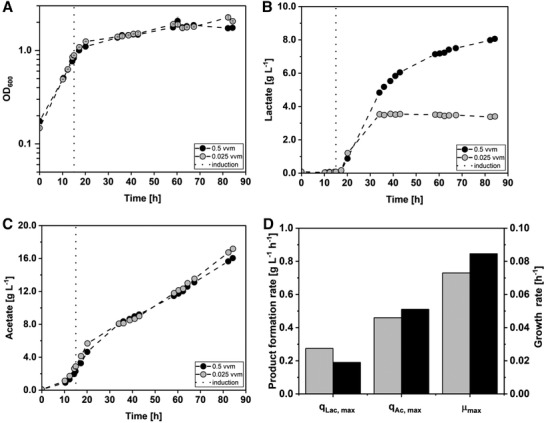
Stirred‐tank batch cultivations of *A. woodii* [P_bgaL_ *_ldhD*_NFP] with different H_2_/CO_2_ gassing rates. Comparison of cultivation with a gassing rate of 0.5 vvm (black circles) and 0.025 vvm (gray circles). (A) Optical cell density (OD_600_); (B) concentration of lactate measured in the culture; (C) concentration of acetate measured in the culture; (D) maximum lactate formation rate (q_lac, max_), maximum acetate formation rate (q_ac, max_) and maximum growth rate (μ_max_). The dotted black line indicates the time point of induction with 1 mM lactose. (T = 30°C; pH = 7.0; P V^–1^ = 6.8 W L^–1^; F_Gas_ = 0.5/0.025 vvm (70:30 H_2_:CO_2_); V_0_ = 1.0 L)

To compare the AiO batch process with a conventional gas fermentation process, *A. woodii* [P*_bgaL_* _*ldhD*_NFP] was cultivated with two different gassing rates of 0.5 and 0.025 vvm of a H_2_/CO_2_ gas mixture (70%/30%) in a stirred‐tank bioreactor. The results are shown in Figure 2. Both batch cultures were inoculated with an initial OD_600_ of 0.17 ± 0.02. The maximum growth rate was reached in both batch processes during the first 15 h of the fermentation. The cells with the higher gassing rate reached a μ_max_ of 0.07 h^–1^ (t = 13.9 h) while the μ_max_ of the cells with the lower gassing rate added up to 0.08 h^–1^ (t = 13.8 h). After 15 h both batch cultures were induced for lactate production with 1 mM lactose. Subsequently, the growth rates declined by a factor of 4.5 to an average value of 0.0174 ± 0.0003 h^–1^. The cells with the higher gassing strategy reached a maximum OD_600_ of 2.1 ± 0.2 after 60.3 h with growth declining afterward. The batch culture with the gassing rate of 0.025 vvm seemed to keep growing up until 82.3 h when the maximum OD_600_ of 2.3 ± 0.1 was reached. The initial and maximum lactate formation rate of both cultures was recorded shortly after induction. The higher gassed culture reached a q_Lac, max_ of 0.27 g L^–1^ h^‐1^ (t = 26.7 h), while the maximum lactate formation rate of the lower gassed culture added up to 0.19 g L^–1^ h^–1^ (t = 25.7 h). After a deceleration phase in lactate formation of the culture with the higher gassing rate between 34 and 58 h process time, q_Lac_ was reduced 6.8‐fold to 0.04 g L^–1^ h^–1^. The final lactate concentration added up to 8.1 g L^–1^. The lactate formation of the culture with the lower sparging rate stopped after 34 h of fermentation time. The maximum value of lactate added up to 3.4 g L^–1^.

Acetate started accumulating in both batch processes after 10 h of process time. The maximum acetate formation rates resulted in 0.5 g L^–1^ h^–1^ (t = 14.7 ± 0.8 h) for both cultivations. After 38 h of process time, q_Ac_ declined in both processes to 0.2 g L^–1^ h^–1^. The maximum acetate concentration measured in the batch process with 0.5 vvm sparging rate added up to 16.1 g L^–1^, while the culture with 0.025 vvm sparging rate reached 17.2 g L^–1^ of acetate. The total amount of H_2_, which was taken up by the microorganisms, added up to 1,345.3 mmol in the fermentation with the higher gassing rate and to 1,188.3 mmol in the cultivation with the lower gassing rate. The overall uptake of CO_2_ was added up to 798.0 mmol in the batch process with 0.5 vvm as sparging rate and to 665.7 mmol in the fermentation with 0.025 vvm as sparging rate. Carbon balances were closed as listed in Table 2.

### 3.3 Synthetic co‐culture of *A. woodii* and *C. drakei*

**FIGURE 3:**
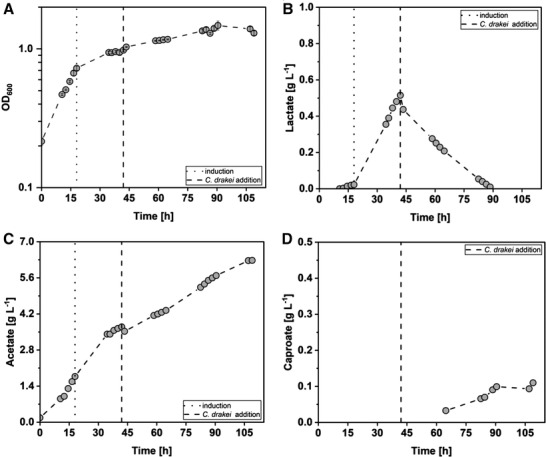
Co‐culture fermentation in a batch‐operated stirred‐tank reactor with A. woodii [P_*bgaL*__*ldhD*_NFP] and *C. drakei*. (A) Optical cell density (OD_600_); (B) concentration of lactate measured in the culture; (C) concentration of acetate measured in the culture; (D) concentration of caproate measured in the culture. The dotted black line indicates the time point when the *A. woodii* [P_*bgaL*__*ldhD*_NFP] culture was induced with 1 mM lactose. The dashed black line indicates the addition of *C. drakei*. (T = 30°C; pH = 7.0; P V^–1^ = 2.4–4.6 W L^–1^; F_CO2_ = 0.9 L h^–1^; I_AiO_ = 600 mA; V_0_ = 1.4 L)

For the establishment of the synthetic co‐cultivation with the AiO‐electrode and CO_2_ sparging in a batch process, *A. woodii* [P*_bgaL_* _*ldhD*_NFP] was first cultivated until it had reached the stationary growth phase. Then, 83 mL of a *C. drakei* bottle culture (OD_600_ = 1.8 ± 0.7) were added to the bioreactor. The co‐cultivation was reproduced (*n* = 2) and showed comparable performances (refer to Figure S2 and Table S5 for repetition data). As shown in Figure 3, the OD_600_ of the *A. woodii* [P*_bgaL_* _*ldhD*_NFP] strain started increasing rapidly after inoculation, reaching a maximum growth rate of 0.037 ± 0.004 h^–1^ (t = 13.8 ± 2.5 h). After the induction of the *A. woodii* [P*_bgaL_* _*ldhD*_NFP] strain at an OD_600_ of 0.75 ± 0.02, the growth rate declined by a factor of 3 to 0.012 ± 0.002 h^–1^. Lactate concentration started increasing after induction, reaching a maximum lactate formation rate of 0.020 ± 0.009 g L^–1^ h^–1^ (t = 33.9 ± 5.8 h) and a maximum concentration of 0.42 ± 0.14 g L^–1^ after 42 h. At this time point, *C. drakei* was added at an OD_600_ of 0.98 ± 0.04. The combined OD_600_ of both microorganisms increased slowly during the cocultivation, reaching a maximum OD_600_ of 1.38 ± 0.24. After the addition of *C. drakei*, the lactate concentration started to decline due to the consumption by *C. drakei*. The maximum lactate consumption rate added up to 0.013 ± 0.003 g L^–1^ h^–1^ (t = 55.8 ± 8.1 h). All lactate produced by *A. woodii* [P*_bgaL_* _*ldhD*_NFP] was consumed by *C. drakei* after 76.4 ± 17.1 h. Caproate concentrations were detected after 64.5 h, and afterward, the concentration started increasing slowly with a maximum formation rate of 0.004 ± 0.001 g L^–1^ h^–1^ (t = 87.4.6 ± 0.1 h). After all lactate was consumed, the caproate concentration stayed constant for the rest of the fermentation. The maximum produced caproate concentration added up to 0.11 ± 0.01 g L^–1^ (t = 98.8 ± 14.5 h).

Acetate formation started directly after inoculation of *A. woodii* [P*_bgaL_* _*ldhD*_NFP]. The maximum acetate formation rate of 0.12 ± 0.03 g L^–1^ h^–1^ was reached during the first 14.6 ± 1.3 h of the fermentation. After the addition of *C. drakei*, the acetate formation rate declined briefly, before continuing to increase, reaching the maximum acetate concentration of 5.62 ± 0.93 g L^–1^ after 106.5 ± 0.7 h.

The total uptake of H_2_ added up to 466.5 ± 91.7 mmol while the overall uptake of CO_2_ was 225.3 ± 48.0 mmol. The AiO‐electrode showed a maximum H_2_ production rate of 9.5 ± 1.2 mmol L^–1^ h^–1^ during the cocultivation. As listed in Table 2, carbon balances were closed within the estimation error of 5%.

### 3.4 Metabolic flux analysis of *A. woodii* [P*_bgaL__ldhD_*NFP]

**FIGURE 4:**
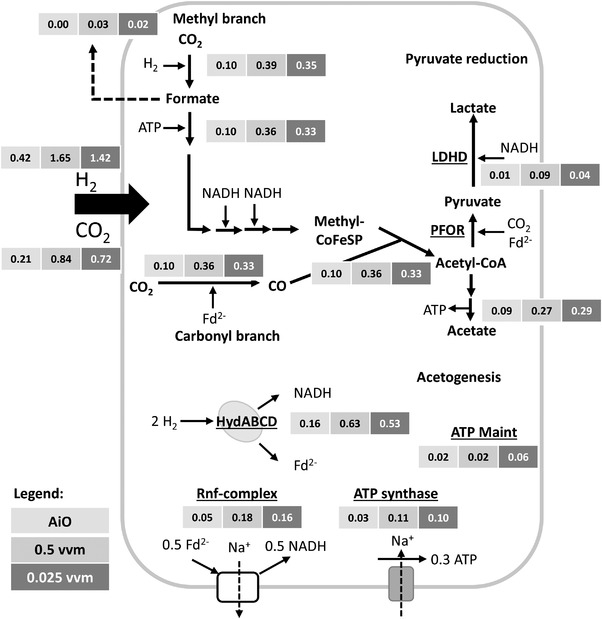
Simplified intracellular stoichiometric network of *A. woodii* [P*_bgaL__ldhD*_NFP] and flux distribution calculated with CellNetAnalyzer. The central metabolism of *A. woodii* [P*_bgaL_* _*ldhD*_NFP] with Wood–Ljungdahl pathway split into methyl‐ and carbonyl‐branch, NADH dependent pyruvate reduction and acetogenesis as well as energy‐coupling reactions catalyzed by the Rnf‐complex, HydABCD and ATP synthetase. Results of product‐based endpoint modeling for the fermentations with the AiO‐electrode (light gray), direct gassing at 0.025 vvm (gray), and 0.5 vvm (dark gray) are given as fluxes (mol L^–1^ h^–1^). ATP Maint denotes ATP which is produced but not needed for the given reactions and as such available for maintenance and further metabolism. CoFeSp, corrinoid iron sulfur protein; LDHD, D‐lactate dehydrogenase; PFOR, pyruvate:ferredoxin oxidoreductase; HydABCD, [FeFe]‐hydrogenase; Rnf, ferredoxin:NAD^+^ oxidoreductase; Fd^2–^, reduced ferredoxin

The estimation of carbon fluxes, by considering final concentrations of acetate, lactate, and peak concentration of formate, derived from the *A. woodii* [P*_bgaL__ldhD_*NFP] AiO‐process and the conventional gas fermentations, were performed with the modified *A. woodii* core model to determine the stoichiometric H_2_/CO_2_ demand. The resulting fluxes are depicted in Figure 4. The underlying assumptions of the constraint‐based approach allow no prediction regarding dynamics or actual intracellular concentrations. However, the substrate fluxes can be converted to respective amounts and compared to the measured H_2_/CO_2_ which was consumed throughout the three fermentations. In each case a H_2_/CO_2_ ratio near 2 was reached, representing the applied gas composition. The calculation of consumed CO_2_ was accurately estimated for the conventional sparging gas. The stochiometric C‐balance of the model is reasonable and fits well with the C‐balance given in Table 2 with a divergence of only 1%–2%. However, the corresponding H_2_‐balance diverged from the presented data in Table 2 (n_H2, total_). The constraint‐based model overestimated H_2_ consumption by 12% for the 0.5 vvm gas sparging fermentation and by 9% for the 0.025 vvm gas sparging fermentation.

## 4 DISCUSSION

The batch experiments with different *A. woodii* strains growing with H_2_ generated by the AiO‐electrode showed that the electrode provided sufficient H_2_ for cell growth and product formation. The *A. woodii* WT showed a 1.7‐fold higher maximum OD_600_ value and a 2‐fold higher growth rate than the cultivations with the *A. woodii* P*_tet__ldhD*_CI_ strain. However, in comparison to the *A. woodii* [P*_bgaL_* _*ldhD*_NFP] strain, the *A. woodii* WT showed only a 1.2‐fold increase in maximum OD_600_ with a very similar μ_max_. More importantly, *A. woodii* [P*_bgaL_* _*ldhD*_NFP] showed a 1.3‐fold increase in overall lactate yield in comparison to the *A. woodii* P*_tet__ldhD*_CI_ strain. This suggests that the recently made adaptions for the *A. woodii* [P*_bgaL_* _*ldhD*_NFP] strain as mentioned by Mook and Beck et al. prove more suitable for the AiO process. Furthermore, the recombinant strains were able to accumulate lactate over the course of their respective fermentations. Note *A. woodii* [P*_bgaL_* _*ldhD*_NFP] was cultivated for 60.4 h and OD_600_ peaked at 42.2 h, afterward no further lactate production or consumption was expected as reported earlier [10]. The *A. woodii* WT strain consumed the previously produced lactate. The reason for this lactate consumption by the *A. woodii* WT strain can be found in the native electron confurcating Ldh/EtF complex, allowing for consumption of lactate while oxidizing Fd^2–^ and generating NADH and pyruvate. Furthermore, the cultivation of *A. woodii* [P*_bgaL_* _*ldhD*_NFP] in H_2_/CO_2_ gas fermentation processes with different sparging rates showed a very high potential for lactate production, reaching 8.1 g L^–1^ which accounts for a 4.1‐fold increase in lactate titers compared to autotrophic serum bottle experiments [10]. To the authors’ best knowledge, this is the highest lactate concentration produced by an autotrophic batch fermentation process reported up to date. In the batch cultivation with direct H_2_ sparging, *A. woodii* [P*_bgaL_* _*ldhD*_NFP] also showed similar growth rates and maximum OD_600_ compared to published data for comparable autotrophic *A. woodii* WT cultivations [9, 32]. The conventional gas fermentation with *A. woodii* [P*_bgaL_* _*ldhD*_NFP] and a H_2_ sparging rate of 0.025 vvm compared to the AiO process showed a 1.8‐fold increase in maximum OD_600_, a 4.3‐fold increase in maximum growth rate and a 6.7‐fold higher lactate titer than the fermentation with the AiO‐electrode. However, the AiO‐electrode generated only 7.7 ± 2.2 mmol L^–1^ h^–1^ of H_2_ during the batch fermentations studied, despite the rather high current density of 8 mA cm^–2^ compared to other BES set ups [23]. The cultivation with a H_2_ sparging rate of 0.025 vvm provided 60.2 mmol L^‐1^ h^–1^ H_2_. Comparable cultivations in literature even reached gassing rates of 179 up to 1,105 mmol L^‐1^ h^‐1^ H_2_ [9, 32, 33]. The cultivation with a gas flow rate of 0.5 vvm (971.0 mmol L^–1^ h^–1^ H_2_) resulted in a 2.4‐fold increase in lactate concentration compared to the lower gassed batch process. This data strongly suggests that H_2_ supply is the main limiting factor in the AiO process. This is supported by the data from the fermentation with the lower H_2_ flow rate of 60.2 mmol L^–1^ h^–1^, which was already limited in lactate production, as the increase of lactate concentration stopped at 3.4 g L^–1^ after only 34 h of process time while the lactate concentration in the fermentation with 971.0 mmol L^–1^ h^–1^ H_2_ kept increasing until the end of the process.

To theoretically match the H_2_ formation rates of the process with direct H_2_ sparging, the electrical current would have to be increased by a factor of 7.9. This in turn would increase the current density to 62.9 mA cm^–2^, if the working electrode surface area would remain the same. Most publications with BES have reported lower current densities [23], and it is uncertain if such high current density would be feasible. Therefore, maintaining the current density of 8 mA cm^–2^ while obtaining higher H_2_ production rates, implies increasing the area of the working electrode by a factor of 7.9. An increase of cathode surface is technically absolutely feasible given that some BES have reported even higher surface areas than would be necessary in this case. The AiO‐electrode has a specific cathode area to liquid volume of 5.3 m^2^ m^–3^, however, some microbial electrolysis cells reach specific values from 6 [34] up to 810 m^2^ m^–3^ [35]. The cell potential of 4.2 ± 0.3 V is also rather high when compared to similar set ups for water electrolysis [23]. Therefore, redesigning the AiO‐electrode by reducing faradaic losses (e.g., by using different separator materials to reduce cathodic O_2_ reduction) and increasing the surface area would possibly decrease the cell potential while increasing its potential for lactate production from H_2_. Besides, using H_2_ gas generated from fossil sources as still done in overwhelming amounts is no alternative for a true carbon‐negative process we propose in this study.

The previously published *A. woodii* core model [30], was modified to include the recombinant NADH‐dependent lactate production pathway. The resulting model (118 reactions) was validated with laboratory data of the three monoculture fermentations with different gassing strategies presented in this study and was reasonably accurate. While the C‐balances fit well with the measured data, there is a deviation between the model and the wet lab data concerning the H_2_‐balance, most likely due to the challenge of measuring the volatile H_2_. However, as already published, the model can be used to determine the potential gas composition and metabolic pathways [7, 36]. While lactate production is currently a limiting step in the co‐cultivation, new insights for improvement of the lactate/acetate ratios can be derived by using respective flux models. Further on, the coupling of the model to growth parameters and subsequent integration of a *C. drakei* core model would be helpful for the determination of seeding densities for co‐cultivation.

The proposed synthetic coculture process for caproate production from CO_2_ and in situ generated H_2_ was proven successful and feasible. The produced lactate by *A. woodii* [P*_bgaL_* _*ldhD*_NFP] was shown to be sufficient to sustain caproate production at low concentrations with comparable lactate production rates to the monocultures presented before. For future optimization of caproate production, the H_2_ availability needs to be increased, which in turn should lead to higher lactate concentrations as well as more NADH needed for chain‐elongation. Longer fermentation times should also be considered. The caproate yield from lactate added up to 0.3 g g^–1^, which is only slightly lower than the yield reported by Zhu et al. (0.4 g g^–1^ [37]). There were no signs of negative interaction between the two species, given that the caproate production correlated with the lactate consumption after *C. drakei* inoculation. However, cell differentiation (e.g., via fluorescence tagging such as FISH [38]) should be implemented for future experiments to monitor the growth of each strain individually. To enhance this coculture process furthermore, the AiO‐electrode could be coupled with an online lactate measurement system to control the H_2_ generation on‐demand. This could reduce the needed H_2_ to a minimum, making the process even more energy efficient. Furthermore, once higher overall caproate titers are reached, an in situ product removal technique such as pertraction [39] will be necessary to prevent inhibitory concentrations of caproate in the medium.

## CONFLICT OF INTEREST

The authors have declared no conflicts of interest.

## NOMENCLATURE

**Table:** 

c_Ac_	[g L^–1^]	Acetate concentration
c_But_	[g L^–1^]	Butyrate concentration
c_Cap_	[g L^–1^]	Caproate concentration
C_i_^*^	[‐]	Number of carbon atoms of a product compound i
C_j_^*^	[‐]	Number of carbon atoms of a substrate compound j
c_Lac_	[g L^–1^]	Lactate concentration
c_Form_	[g L^–1^]	Formate concentration
E_cell_	[V]	Cell potential of the AiO‐electrode
F_CO2_	[L h^–1^]	CO_2_ sparging rate
F_Gas_	[vvm]	Gas sparging rate
I_AiO_	[mA]	Electrical current of the AiO‐electrode
*j*	[mA cm^–2^]	Current density
n_CO2_	[mol]	Total CO_2_ uptake
n_H2_	[mol]	Total H_2_ uptake
n_i_	[mol]	Amount of a product compound i
n_j_	[mol]	Amount of a substrate compound j
P	[W]	Power output
P V^‐1^	[W L^–1^]	Volumetric power input
q_Ac_	[g L^–1^ h^–1^]	Acetate formation rate
q_Cap_	[g L^–1^ h^–1^]	Caproate formation rate
‐q_Lac_	[g L^–1^ h^–1^]	Lactate consumption rate
q_Lac_	[g L^–1^ h^–1^]	Lactate formation rate
*Greek symbols*		
μ_max_	[h^–1^]	Maximum cell growth rate

## Supporting information



Supporting Information

## Data Availability

The data that support the findings of this study are openly available in Mendeley Data at doi: https://doi.org/10.17632/7nf9hjwf43.3.
