# Supplementary material for: Novel synthetic co‐culture of Acetobacterium woodii and Clostridium drakei using CO2 and in situ generated H2 for the production of caproic acid via lactic acid
Source: Eng Life Sci. 2022 May 22;23(1):e2100169. doi: 10.1002/elsc.202100169 (PMC9815077; doi:10.1002/elsc.202100169)
Supplement: Supplementary file 1 — Supporting Information [file ELSC-23-e2100169-s001.pdf]

Supplement Material

Table S1: Composition of the modified DSMZ medium 135 for serum bottle fermentations.

Component	Concentration [g L^{-1}]
NH_4Cl	0.2
KH_2PO_4	1.8
K_2HPO_4	8.4
Yeast extract	6.0
Cysteine-HCl x H_2O	0.3
$NaHCO_3$	10.0
	**Concentration [mL L^{-1}]**
Resazurin solution (0.1 %)	1.0
Trace elements solution (see Table S2)	2.0
Na_2S solution (15 g L^{-1})	20.0
Vitamin solution (see Table S3)	2.0
$MgSO_4$ x 7 H_2O solution (165 g L^{-1})	2.0
Selenite-tungsten solution (see table S4)	1.0

Table S2: Composition of the SL9 trace elements solution (Tschech and Pfennig, 1984) used in all fermentation experiments.

Component	Concentration [g L^{-1}]
Nitrilotriacetic acid	12.8
$MnCl_2$ x 2 H_2O	0.1
$FeCl_2$ x 4 H_2O	2.0
$CoCl_2$ x 6 H_2O	0.2
	**Concentration [mg L^{-1}]**
$ZnCl_2$	70.0
$CuCl_2$ x 2 H_2O	2.0
H_3BO_3	6.0
Na_2MoO_4 x 2 H_2O	36.0
$NiCl_2$ x 6 H_2O	24.0

Table S3: Composition of the vitamin solution (Wolin et al., 1963 mod.) used in all fermentation experiments.

**Component**	**Concentration [mg L^{-1}]**
Biotin	25.0
Folic acid	25.0
Pyridoxine-HCl	50.0
Thiamine-HCl	50.0
Riboflavin	50.0
Nicotinic acid	50.0
D-Ca-Pantothenate	50.0
Cyanocobalamine	25.0
α-aminobenzoic acid	50.0
Lipoic acid	25.0

Table S4: Composition of the selenite-tungsten solution (Tschech and Pfennig, 1984) used in all fermentation experiments.

**Component**	**Concentration [mg L^{-1}]**
NaOH	500.0
Na_2SeO_3 x 5 H_2O	3.0
Na_2WO_4 x 2 H_2O	4.0

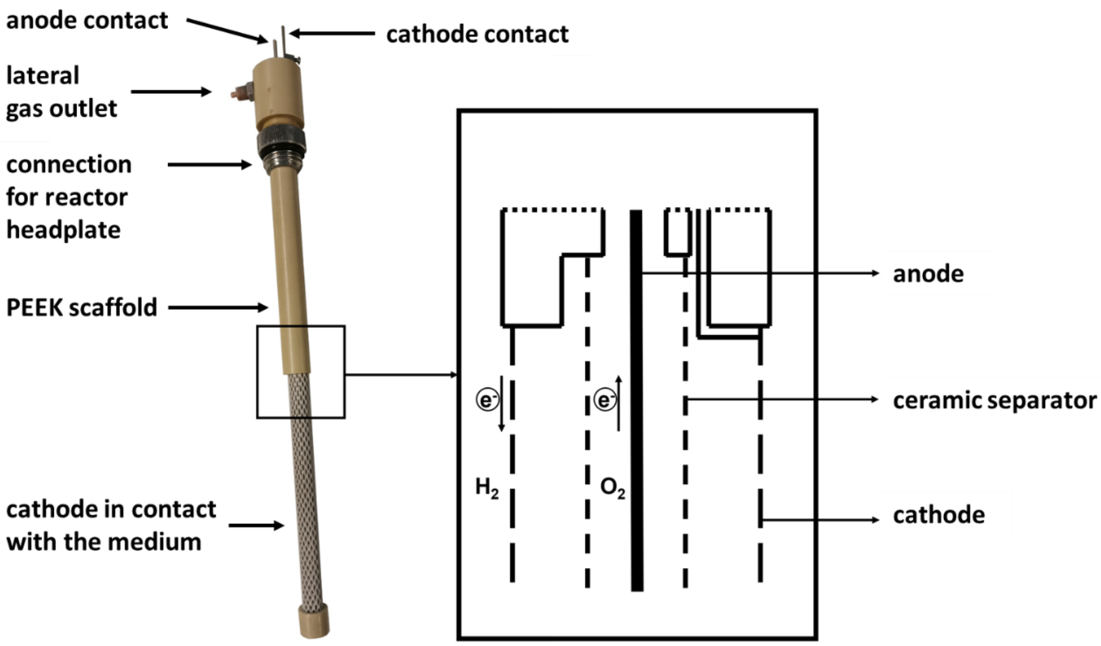

Figure S1: Simplified scheme of the All-in-One electrode for in-situ electrolysis (adapted from Utesch and Zeng, 2018)

Equation S1: example calculation for carbon balance of *A. woodii* [P$_{bgaL}$_*ldhD*_NFP] fermentation with AiO-electrode (see chapter 3.1)

$$C_{recovery} = \frac{\sum_i^n n_i \cdot C_i^*}{\sum_j^n n_j \cdot C_j^*} = \frac{n_{Lac} \cdot C_{Lac}^* + n_{Form} \cdot C_{Form}^* + n_{Ac} \cdot C_{Ac}^* + n_{BM} \cdot C_{BM}^*}{n_{CO_2} \cdot C_{CO_2}^* + n_{NaHCO_3} \cdot C_{NaHCO_3}^*}$$

$$= \frac{(5.4\, mmol \cdot 3 + 7.7\, mmol \cdot 1 + 73.2\, mmol \cdot 2 + 4.5\, mmol \cdot 4)}{(99.7\, mmol \cdot 1 + 82.4\, mmol \cdot 1)} = 1.03$$

C_{Ac} = number of carbon atoms of acetate

C_{BM} = number of carbon atoms of biomass

C_{CO2} = number of carbon atoms of CO_2

C_{Form} = number of carbon atoms of formate

C_{NaHCO3} = number of carbon atoms of $NaHCO_3$

C_{Lac} = number of carbon atoms of lactate

n_{Ac} = amount of acetate generated during the fermentation

n_{BM} = amount of biomass generated during the fermentation

n_{CO2c}= amount of CO_2 consumed during the fermentation

n_{Form} = amount of formate generated during the fermentation

n_{NaHCO3} = amount of $NaHCO_3$ at the beginning of the fermentation

n_{Lac} = amount of lactate generated during the fermentation

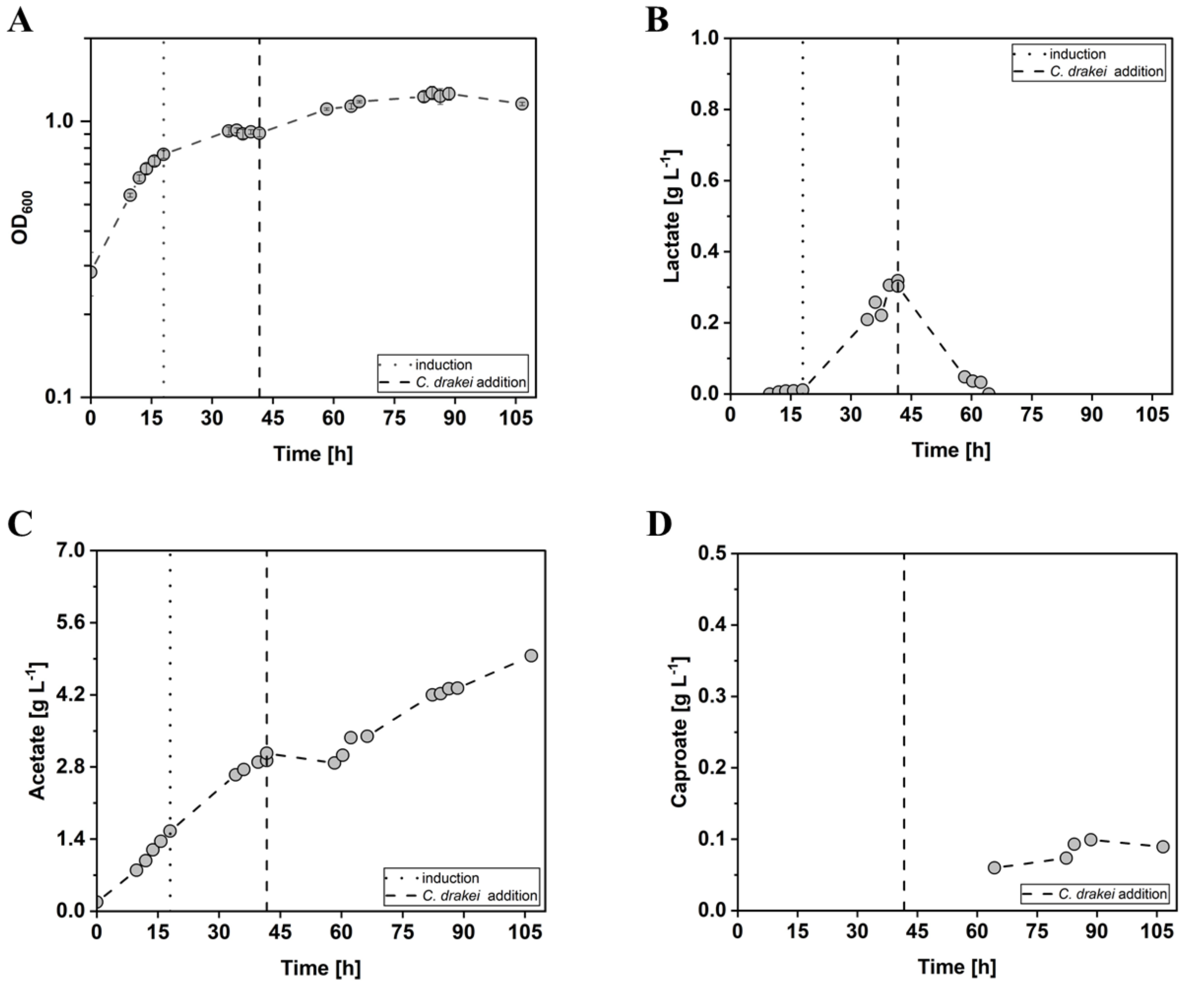

Figure S2: Replicate co-culture fermentation in a batch operated stirred-tank reactor with *A. woodii* [P*bgaL*_*ldhD*_NFP] and *C. drakei*. (A) optical cell density (OD_{600}); (B) concentration of lactate measured in the culture; (C) concentration of acetate measured in the culture; (D) concentration of caproate measured in the culture. The dotted black line indicates the time point when the *A. woodii* [P*bgaL*_*ldhD*_NFP] culture was induced with 1 mM lactose. The dashed black line indicates the addition of *C. drakei*. (T = 30 °C; pH = 7.0; $P V^{-1}$ = 2.4 – 4.6 W L^{-1}; F_{CO2} = 0.9 L h^{-1}; I_{AiO} = 600 mA; V_0 = 1.4 L)

Table S5: Maximum product formation and consumption rates, maximum cell density and growth rate, maximum product concentrations, total gas uptake and carbon-balances of replicate co-culture fermentation in a batch operated stirred-tank reactor with *A. woodii* [P*bgaL*_*ldhD*_NFP] and *C. drakei*.

**Parameter**	OD_{max}	μ_{max}	$c_{Lac,max}$	$q_{Lac,max}$	$-q_{Lac,max}$	$c_{Ac,max}$	$c_{Cap,max}$	$q_{Cap,max}$	$c_{Form,max}$	$c_{But,max}$	$n_{CO2,total}$	$n_{H2,total}$
**Unit**	-	h^{-1}	g L^{-1}	g L^{-1} h^{-1}	g L^{-1} h^{-1}	g L^{-1}	g L^{-1}	g L^{-1} h^{-1}	g L^{-1}	g L^{-1}	mol	mol
**Value**	1.27	0.03	0.32	0.01	0.02	4.96	0.10	0.01	0.30	0.32	0.19	0.40

References

Tschech, A., Pfennig, N., Growth yield increase linked to caffeate reduction in *Acetobacterium woodii*. *Arch. Microbiol.* 1984, *137*, 163–167. doi: 10.1007/BF00414460

Utesch T., Zeng A-P., A novel all-in-one electrolysis electrode and bioreactor enable better study of electrochemical effects and electricity-aided bioprocesses. *Eng. Life Sci*. 2018, *18*, 600–610. doi: 10.1002/elsc.201700198

Wolin, E. A., Wolin, M. J., Wolfe, R. S., Formation of methane by bacterial extracts. *J. Bio. Chem.* 1963, *238*, 2882–2886. doi: 10.1016/S0021-9258(18)67912-8
